# Chronically ill patients’ preferences for a financial incentive in a lifestyle intervention. Results of a discrete choice experiment

**DOI:** 10.1371/journal.pone.0219112

**Published:** 2019-07-25

**Authors:** Claudia Molema, Jorien Veldwijk, Wanda Wendel-Vos, Ardine de Wit, Ien van de Goor, Jantine Schuit

**Affiliations:** 1 Tilburg University, Department of Tranzo, Scientific Center for Care and Welfare, Tilburg, the Netherlands; 2 National Institute for Public Health and the Environment, Centre for Nutrition, Prevention and Health Services, Bilthoven, the Netherlands; 3 Centre for Research Ethics and Bioethics, Uppsala University, Uppsala, Sweden; 4 University Medical Center Utrecht, Julius Center for Health Sciences and Primary Care, Utrecht, the Netherlands; 5 VU University, Department of Health Science and EMGO institute for Health and Care Research, Amsterdam, the Netherlands; BITS Pilani, INDIA

## Abstract

**Background:**

The preferences of diabetes type 2 patients and cardiovascular disease patients for a financial incentive added to a specified combined lifestyle intervention were investigated.

**Methods:**

A discrete choice experiment questionnaire was filled out by 290 diabetes type 2 patients (response rate 29.9%). Panel-mixed-logit models were used to estimate the preferences for a financial incentive. Potential uptake rates of different financial incentives and relative importance scores of the included attributes were estimated. Included attributes and levels were: form of the incentive (cash money and different types of vouchers), value of the incentive (ranging from 15 to 100 euros), moment the incentive is received (start, halfway, after finishing the intervention) and prerequisite for receiving the incentive (registration, attendance or results at group or individual level).

**Results:**

Prerequisites for receiving the financial incentive were the most important attribute, according to the respondents. Potential uptake rates for different financial incentives ranged between 37.9% and 58.8%. The latter uptake rate was associated with a financial incentive consisting of cash money with a value of €100 that is handed out after completing the lifestyle program with the prerequisite that the participant attended at least 75% of the scheduled meetings.

**Conclusions:**

The potential uptake of the different financial incentives varied between 37.9% and 58.8%. The value of the incentive does not significantly influence the potential uptake. However, the potential uptake and associated potential effect of the financial incentive is influenced by the type of financial incentive. The preferred type of incentive is €100 in cash money, awarded after completing the lifestyle program if the participant attended at least 75% of the scheduled meetings.

## Introduction

Physical inactivity and a poor diet contribute to the development of a range of chronic diseases and explain part of the variation in premature mortality [1, 2]. Many people do not meet the standards for physical activity levels developed by the World Health Organization and are physically inactive [2, 3]. Patients with diabetes mellitus type 2 or with coronary heart disease are groups with relatively high prevalence of physical inactivity [2].

Health care providers seek effective ways to change this unhealthy behavior. One way to do so is by offering (chronically ill) patients a lifestyle program that includes physical activity and improving eating behavior, called combined lifestyle interventions (CLIs) [4, 5]. However, participation rates in lifestyle programs vary considerably. Some programs have good participation rates, others struggle with low participation rates. For example, the participation rates of diabetes mellitus type 2 patients in lifestyle programs, mainly implemented in primary care, range from 10% to 80% and multiple studies mentioned that boosting the motivation of participants requires more attention [6–9].

Health promoting financial incentives (HPFI) might increase patients’ participation rates and adherence to lifestyle programs and are increasingly implemented by public authorities and health insurance companies to promote healthy behaviors [10–13]. However, the effectiveness of financial incentives added to lifestyle programs in the health care setting for individuals is still inconclusive [14, 15]. HPFI are cash or cash-like rewards or fines, provided contingent on (non-) performance of healthy behaviors. The two main categories are positive (e.g. reward or discount) and negative (e.g. a fine or a higher contribution to the lifestyle program or health insurance premium) incentives [16]. Within these two categories, the incentive can vary on different characteristics. For example, they can vary in value, the moment that the participants receive their incentive (before the intervention or afterwards), conditions that have to be fulfilled to receive the incentive, and many more characteristics (e.g. provider of the incentive, lottery system or guaranteed reward). The incentive can be targeted at the participation rate, at compliance with instructions, or at outcome measures such as a higher physical activity level, a healthier diet or weight loss.

A financial incentive is an extrinsic motivation. A well-known argument for not using financial incentives is the crowding-out effect. This refers to the mechanism that extrinsic motivation in the form of financial incentives might undermine and replace the intrinsic motivation. However, in the field of health related behavior, so far no evidence has been found to support this possibility [17, 18]. A plausible explanation is that individuals eligible for a CLI do not have any intrinsic motivation to change their health behavior. Therefore, intrinsic motivation cannot be replaced by extrinsic motivation. By adding an extrinsic motivation to start participating in a CLI, participants may develop intrinsic motivation during the course of the program, for example because they develop a better physical condition.

To prevent the implementation of an ineffective or even counterproductive HPFI, insight into the preferences of the target population with regard to the HPFI is of crucial importance. To date, in the design phase of a new intervention that includes a financial incentive, hardly any research (if any) has been performed into the target populations’ preferences regarding the characteristics of the financial incentive. Previous studies do however provide some general information about preferences regarding incentives. For example, the study by Gneezy et al. shows that if a financial incentive is not high enough, it might justify or even promote undesirable behavior [19]. The study by Barte et al. shows that there is a need for more insight into the effectiveness of the different types and components of a financial incentive and that for example unconditional financial incentives do not affect physical activity [20].

One way to determine preferences with regard to HPFI is by performing a Discrete Choice Experiment (DCE). This is a quantitative technique and a frequently used tool in (public) health research to estimate possible participation rates in interventions or medical treatments and to provide knowledge on the components of the programs that determine the participation rates [21, 22]. The DCE methodology is based on the Random Utility Theory and assumes that any intervention or treatment can be described by its characteristics (i.e. attributes, such as the form of the incentive). In this study, a discrete choice experiment is performed to identify which financial incentive is preferred by diabetes mellitus type 2 patients to be added to a specific lifestyle intervention that aims to improve the participant’s physical activity level and eating habits.

## Material and methods

This study does not fall under the scope of the Dutch Medical Research Involving Human Subjects Act (in Dutch; WMO) and therefore did not need to undergo a review by a Medical Ethical Committee. Since an Institutional Review Board (IRB) approval is only needed when daily life of participants is influenced or participants should perform specific actions an IRB approval was not warranted and therefore not obtained. The data were anonymized prior to the moment that the authors received the data. The authors did not have access to any identifying information. This DCE was conducted as preparatory part of an intervention study aimed at evaluating the efficacy and feasibility of a financial incentive added to a lifestyle intervention. The results of this experiment were used to design the financial incentive that was added to a lifestyle intervention. The lifestyle intervention aimed to improve the participants’ physical activity behavior and eating habits. This lifestyle intervention was designed for patients at least 18 years of age, with diabetes mellitus type 2 and/or cardiovascular disease, who received integrated care in the primary care setting in the region of a care group in the southern part of the Netherlands. In this section, the methods of the DCE are described.

### Study population

The study population for the DCE was part of the study population of the main project and was selected based on a geographic area. The area of the care group was divided into four parts. Three subareas were selected for the intervention study in which the CLI and a financial incentive would be implemented. One subarea was excluded from the intervention study and the patients living in this area were invited to fill out the questionnaire. All selected patients were at least 18 years of age, with diabetes mellitus type 2 and/or cardiovascular disease who receive integrated care in the primary care setting for their diseases. They received the DCE questionnaire by conventional mail, with a reminder sent two weeks after the first mailing. As respondents completed their questionnaire anonymously, no information about non-responders is available.

### Discrete choice experiment

The attributes and levels included in the current study (**Table 1**) were determined in a stepwise manner. First, a list of characteristics of financial incentives was compiled, based on available research literature [11, 23]. This list was discussed in three focus group interviews (eleven participants in total) to ensure that the most important attributes for the decision-making process were included. The focus groups consisted of patients with diabetes mellitus type 2 and/or cardiovascular disease. Since no new attributes were mentioned during the focus groups, the existing list of potential attributes was sent to a new subsample of patients in a different geographical location in the northern part of the Netherlands. We believe the patients of this subsample are comparable to patients in our study as patients in all Dutch care groups receive similar diabetes care, based on Dutch general practitioners’ guidelines. These patients were asked to rank the attributes from most to least important. In total, 30 individuals filled out the ranking forms, of which eleven had participated in the focus group interviews. This process led to the inclusion of four attributes of which one had three levels (moment), two had four levels (form and value) and one had five levels (prerequisite). The levels were chosen based on the feasibility in practice. See Table 1 for the levels and attributes that were included in this DCE.

**Table 1:** Attributes and levels that were included in this DCE.

Attribute	Level
Form of the incentive	Cash money (reference level)
	Voucher exchangeable in numerous stores
	Voucher exchangeable in numerous restaurants
	Voucher for theater- or concert tickets
Value of the incentive	15 euros
	35 euros
	65 euros
	100 euros
Moment incentive is received	At the start of the lifestyle program (reference level)
	After finishing the lifestyle program
	Halfway (50%) and after finishing (50%) the lifestyle program
Prerequisite for receiving the incentive	Registration for the lifestyle program (reference level)
	75% attendance at individual level
	75% attendance at group level
	Individual result of the fitness test* (you will receive the reward if you have a better score at the end of the program than at the start of the program on the fitness test)
	Group result of the fitness test* (you receive the reward if at least 80% (8 out of 10) participants score better at the end of the program than at the start of the fitness test.)

*The fitness test includes measuring Body Mass Index, body fat percentage, waist circumference, maximum hand grip strength, maximum leg press and VO_2_^max^.

#### Study design

A full factorial design with the identified attributes and levels as described in Table 1 would test all possible combinations of attributes and levels and would therefore consist of 240 (3*4*4*5) different scenarios. Due to obvious methodological (bias) and cognitive (burden on participants) reasons, not all these scenarios were included.

After pilot testing our original orthogonal DCE design, NGene 1.0 (ChoiceMetrics, 2011) software was used to develop a D-efficient design, which entails a design with an optimal variance-covariance matrix [24, 25]. The design was restricted because not all combinations of attribute levels are possible in real life. For example, when the reward is given at the start of the intervention the only requirement that can be met is registration for the lifestyle program. Our final design consisted of 18 unique choice tasks. To limit the burden for the respondents, NGene divided these 18 choice tasks into two sets of nine choice tasks and each set was disseminated among half of the study population.

#### Questionnaire

**Fig 1:**
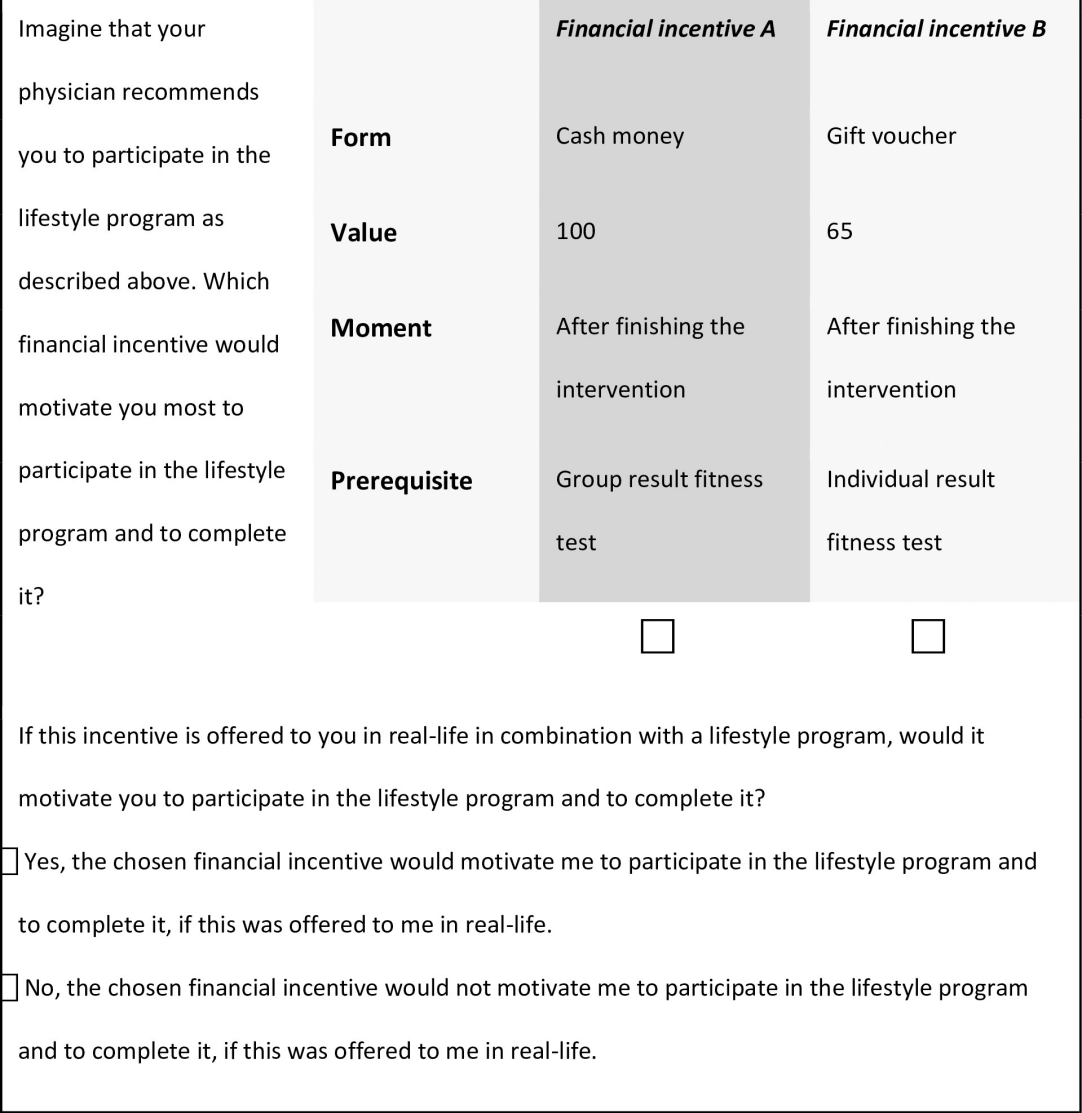
Example of a choice task.

The questionnaire consisted of two parts (S1 and S2 Files). In the first section the participant had to fill out 29 questions about age, gender, socioeconomic status, nationality, physical activity level, eating habits, quality of life (EQ-5D questionnaire; score between 0 and 1), health literacy [26, 27], and attitude towards lifestyle programs. The second part of the questionnaire consisted of the actual DCE. Every respondent was presented a series of choice tasks. These choice tasks consisted of two different financial incentives described by means of varying levels of the included four attributes (**Table 1**). In the questionnaire, definitions for all attributes were specified. Every choice task started with the question: *‘Imagine that your physician recommends that you participate in the lifestyle program as described above*. *Which financial incentive would motivate you most to participate in the lifestyle program and to complete it*?*’* An example of a choice task is shown in **Fig 1**.

Following each of the nine choice tasks, the participant was asked whether the financial incentive of their choice would actually motivate them to participate in and finish the lifestyle program or not (opt-out question). This option was included, because in real life people also have the option not to participate in the program. After completing the nine choice tasks, patients had to fill out six questions about their attitude and opinion regarding financial incentives. Response options were the characteristics of financial incentives in the choice tasks. Questions were asked about their opinion about using financial incentives, whether they believe it could motivate them or other people to work on their health, which attribute is most important in their choice for accepting or declining a financial incentive, and which form and prerequisites they prefer most.

The questionnaire was pilot tested in the development phase to make sure the target group was able to fill out the questionnaire as intended. Respondents of the pilot questionnaire (n = 30) were able to give comments on the choice of words, the length of the questionnaire and the layout, of the final questionnaire. The respondents did not report any lack of clarity, so we did not change the text of the questionnaire.

### Statistical analysis

#### Direct attribute ranking

Before respondents answered the choice tasks, they were asked by means of a multiple-choice question which characteristic (i.e. attribute) of a financial incentive they found most important when choosing to accept or decline a financial incentive. The results of this question are reported as percentages of the respondents who rank a certain attribute as most important.

#### Preferences with regard to the incentive

To estimate the preferences of the target population with regard to a financial incentive, data was analyzed using panel-mixed-logit (Panel-MIXL) models. These models adjust the results for the multilevel structure of the data; every respondent completed nine choice tasks, therefore their answers may be correlated, which is accounted for using these analytical models. The following equation was tested using these models:

U = V + ε = β_0_ + β_1_ * voucher exchangeable in multiple stores + β_2_ * voucher exchangeable in multiple restaurants + β_3_ * voucher for theater- or concert tickets + β_4_ * value + β_5_ * after the lifestyle program + β_6_ * halfway (50%) and after completing (50%) the lifestyle program + β_7_ * 75% attendance at individual level + β_8_ * 75% attendance at group level + β_9_ * individual result fitness test + β_10_ * group result fitness test + ε

V describes the measurable utility of a specific financial incentive based on the attributes that were included in the DCE. β_0_ represents the alternative specific constant and β_1 -_ β_10_ are the attribute level estimates that indicate the relative importance of each attribute. The opt-out option was modelled as having a utility of zero. Finally, ε describes the unmeasured and unmeasurable variation in the respondents’ preferences.

All non-linear variables are coded using effects coding. In contrast to dummy coding, the reference category is coded as -1. The coefficient for the reference category is therefore -1*(sum of the β of the other attribute levels within the same attribute).

Based on the results of the model fit tests (Log Likelihood ratio test and AIC), all attributes were included as random parameters with a normally distributed standard deviation. By doing this, the model accounts for the heterogeneity in respondents’ preferences concerning those attributes.

#### Relative importance scores of the attributes

The relative importance scores of the attributes represent the relative distance of all attributes to the most important attribute on a scale of 0–1. Since the coding of the data influences the estimates of the model, a new model was used to calculate the relative importance scores, in which all attribute levels have been coded similarly (-1 to 1).

The attribute with the highest relative importance score is most decisive in the choice for a financial incentive. To calculate these relative importance scores, first the difference between the largest and the smallest attribute level estimate had to be calculated for each attribute. An importance score of 1 was given to the attribute with the largest difference value. The other relative importance scores were calculated by dividing the difference values by the largest difference value, resulting in a relative distance of all attributes to the most important attribute.

#### Potential uptake of different incentives

The potential uptake of a financial incentive that consists of a specific set of attributes was estimated. Since all attributes were included as random parameters in the analyses and their standard deviation had to be taken into account, simulation was used to calculate the choice probabilities. The mean participation rates of all simulations (n = 10,000) was estimated by taking the average of all simulated participation rate probabilities, which were calculated as 1/(1+exp^-v^).

## Results

### Participant characteristics

The questionnaire was sent to 971 individuals and 290 questionnaires were returned in total (response rate of 29.9%). The mean age of the respondents was 69.4 years (range 38 to 92 years) and 60.4% were male. About half of the participants had a low educational level. Participants scored their health-related quality of life (EQ-5D) on average with a score of 0.84 for men and 0.79 for women (overall score of 0.82), while 12.2% of the respondents had an inadequate health literacy (score ≤2; self-reported). Almost a quarter of the participants believed that using financial incentives to motivate people to improve their health would be useful and 42.7% considered it not useful. In total, 16.9% of the respondents reported that a financial incentive would personally motivate them to improve their health while 64.2% reported that it would not motivate them (**Table 2**).

**Table 2:** General characteristics of the study population (N = 290).

		Mean (SD)	Percentage
Age (n = 279)		69.4 (9.9)	
Gender (n = 283)	Male		60.4
Ethnicity (n = 290)	Dutch		96.2
Educational level (n = 285)	Low		54.4
	Medium		19.1
	High		20.9
	Other		5.5
Household income per month (n = 260)	Less than €1000		8.5
	€1000 to €2000		33.5
	€2000 to €3000		28.5
	€3000 to €4000		16.2
	€4000 to €5000		8.5
	€5000 or more		5.0
Health-related quality of life (EQ5d score)	Overall (n = 275)	0.82 (0.17)	
	Men (n = 167)	0.85 (0.14)	
	Women (n = 108)	0.79 (0.19)	
Health literacy	Health literacy score (range 0–4) (n = 287)	3.4 (0.88)	
	Inadequate health literacy (n = 287)		12.2
Opinion on financial incentives to improve people’s health status (n = 253)	Very useful / Useful		24.1
	Neutral		33.2
	Not very useful / Not useful at all		42.7
Respondents’ opinion whether a financial incentive would motivate them to improve their health (n = 254)	Yes, it would motivate me		16.9
	No, it would not motivate me		64.2
	No opinion		18.9

### Direct attribute ranking

**Fig 2:**
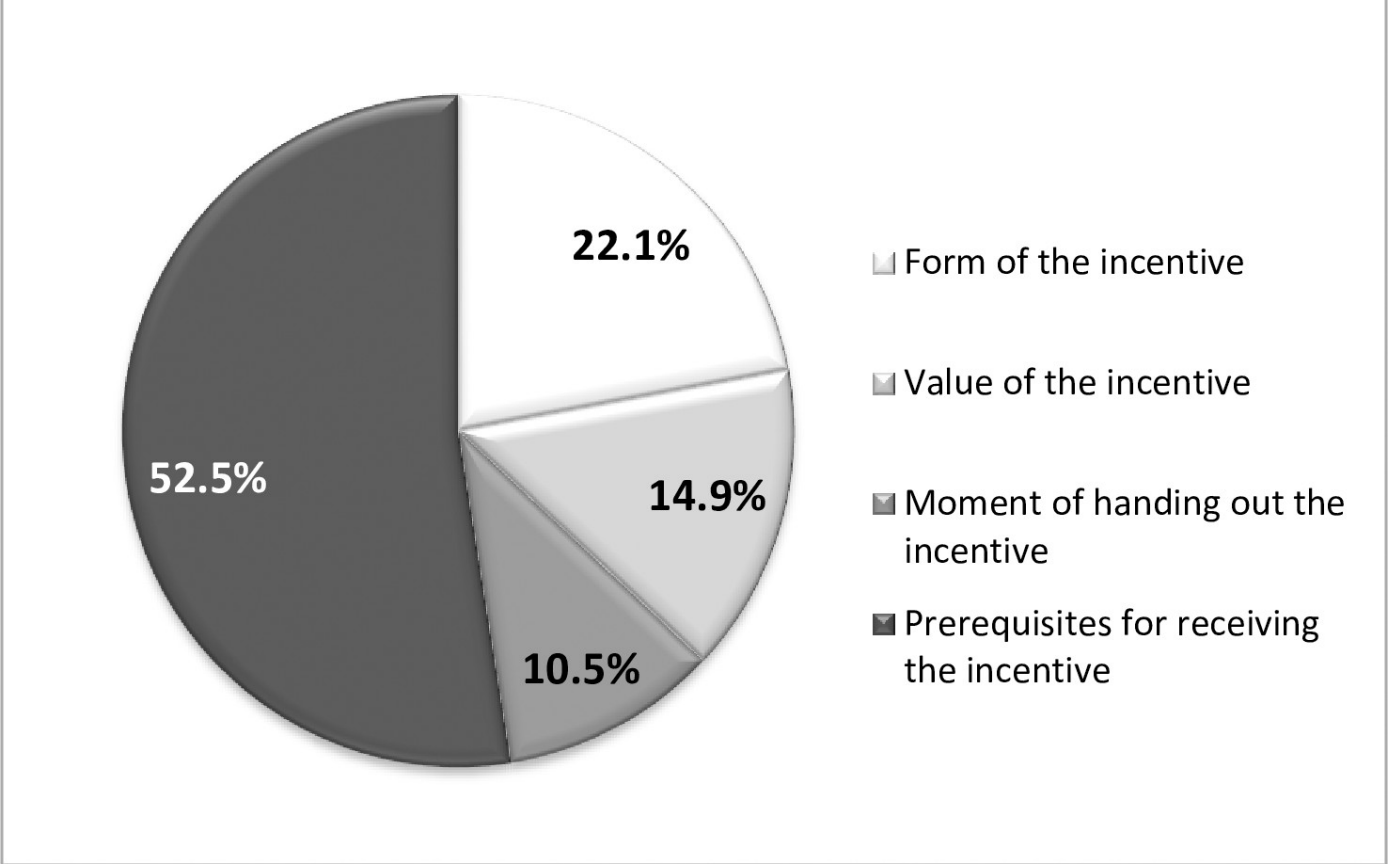
Direct attribute ranking.

Most of the respondents (52.5%) reported that the prerequisites for receiving the incentive were the most important attribute for them, followed by the form of the incentive (22.1%) and the value of the incentive (14.9%). Finally, the smallest number of respondents (10.5%) marked the moment of awarding the incentive as the most important attribute (**Fig 2)**.

### Preferences with regard to the incentive

Respondents preferred cash money over all other forms of incentives, while a voucher for theater or concert tickets was the least preferred. The higher the value of the incentive, the more individuals preferred the incentive. Respondents preferred to receive the incentive after completing the lifestyle program over receiving it at any other point in time. Finally, respondents preferred the prerequisite of 75% attendance at individual level over all other prerequisites. The least preferred prerequisite for receiving the incentive was the group result of the fitness test **(Table 3)**.

**Table 3:** Preferences for a financial incentive.

		Estimate	SE
Constant	Mean	-0.603	0.252
	SD	3.712	0.265
			
*Form of the incentive*			
Cash money (reference)	Mean	0.173	0.098
	SD	0.525	0.136
Voucher exchangeable in numerous stores	Mean	0.052	0.080
	SD	0.058	0.327
Voucher exchangeable in numerous restaurants	Mean	0.143	0.085
	SD	0.228	0.135
Voucher for theater or concert tickets	Mean	-0.368	0.102
	SD	0.470	0.131
			
*Value of the incentive*	Mean	0.243	0.213
	SD	1.248	0.286
			
*Moment the incentive is received*			
At the start of the lifestyle program (reference)	Mean	-0.356	0.047
	SD	0.343	0.127
After completing the lifestyle program	Mean	0.522	0.088
	SD	0.331	0.124
Halfway (50%) and after completing the lifestyle program (50%)	Mean	-0.166	0.118
	SD	0.090	0.154
*Prerequisite for receiving the incentive*			
Registration for the lifestyle program (reference)	Mean	0.006	1.067
	SD	0.644	0.165
75% attendance at individual level	Mean	0.608	0.106
	SD	0.008	0.169
75% attendance at group level	Mean	-0.103	0.110
	SD	0.111	0.144
Individual result of the fitness test	Mean	0.225	0.118
	SD	0.124	0.196
Group result of the fitness test	Mean	-0.736	0.150
	SD	0.622	0.164

### Relative importance scores of the attributes

**Fig 3:**
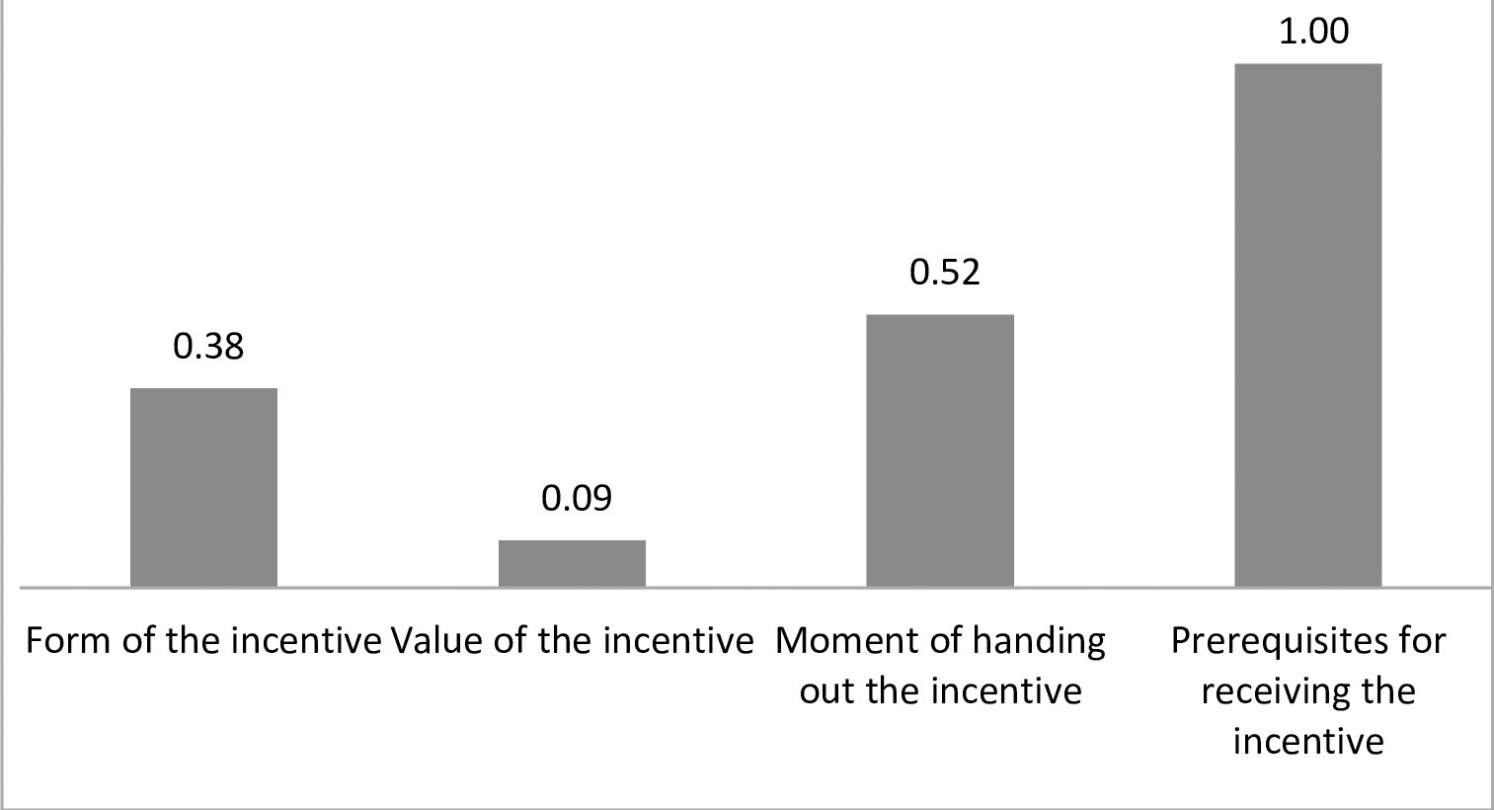
Relative importance scores of the attributes included in the DCE.

Respondents reported that a prerequisite for receiving the incentive was the most important attribute (score 1.00). The moment of receiving the incentive was about half as important (0.52) and the value of the incentive has the lowest relative importance score. **Fig 3** shows the results in more detail.

### Potential uptake of different incentives

Potential uptake rates varied strongly, ranging from 37.9% to 58.8%, based on the characteristics of the incentive. The financial incentive with the highest potential uptake (58.8%) was cash money with a value of €100 that is handed out afterwards with the requirement that the individual has attended at least 75% of the appointments (**Table 4**). The incentive with the lowest potential uptake (37.9%) was a voucher for theater or concert tickets of €15 that is handed out at the start with no requirements besides registration for the lifestyle program (**Table 4**).

**Table 4:** Potential uptake in percentages of all possible financial incentives (lowest and highest potential uptake rates in bold).

	Cash	Voucher exchangeable in multiple stores	Voucher exchangeable in multiple restaurants	Voucher theater or concert tickets
**15 euros**				
At the start + registration lifestyle program	43.1	41.8	42.9	**37.9**
Halfway and after completing the program + 75% attendance individual level	50.5	49.1	50.3	44.8
Halfway and after completing the program + 75% attendance group level	43.7	42.3	43.3	38.6
After completing the program + 75% attendance individual level	57.6	55.7	56.6	51.9
After completing the program + 75% attendance group level	50.5	49.1	50.2	45.0
After completing the program + individual result fitness test	53.5	51.9	53.2	48.6
After completing the program + group result fitness test	44.3	43.7	44.1	39.2
**35 euros**				
At the start + registration lifestyle program	43.8	42.3	43.6	38.5
Halfway and after completing the program + 75% attendance individual level	51.1	49.9	50.7	45.6
Halfway and after completing the program + 75% attendance group level	44.7	43.1	43.5	39.0
After completing the program + 75% attendance individual level	57.8	56.4	57.4	52.0
After completing the program + 75% attendance group level	50.6	49.8	50.5	45.3
After completing the program + individual result fitness test	53.7	52.8	53.7	48.7
After completing the program + group result fitness test	44.7	43.4	44.2	39.9
**65 euros**				
At the start + registration lifestyle program	44.3	43.1	44.0	39.5
Halfway and after completing the program + 75% attendance individual level	51.7	50.3	51.5	46.4
Halfway and after completing the program + 75% attendance group level	45.1	44.1	44.7	40.0
After completing the program + 75% attendance individual level	58.0	57.1	57.6	53.2
After completing the program + 75% attendance group level	51.1	50.1	51.0	46.1
After completing the program + individual result fitness test	54.6	53.4	54.4	49.0
After completing the program + group result fitness test	45.4	44.2	45.5	40.4
**100 euros**				
At the start + registration lifestyle program	45.4	44.1	44.9	40.0
Halfway and after completing the program + 75% attendance individual level	52.5	51.2	52.2	47.5
Halfway and after completing the program + 75% attendance group level	45.8	44.7	45.2	40.7
After completing the program + 75% attendance individual level	**58.8**	57.4	58.2	53.7
After completing the program + 75% attendance group level	52.4	51.0	52.2	48.6
After completing the program + individual result fitness test	55.1	54.2	54.9	50.5
After completing the program + group result fitness test	46.7	45.2	46.1	41.8

## Discussion

We performed a discrete choice experiment to identify which financial incentive should preferably be added to a combined lifestyle intervention among patients with diabetes type 2. This study is, to our knowledge, the first to investigate preferences for a financial incentive added to a lifestyle program.

The most preferred financial incentive resulting in the highest potential uptake based on this DCE was cash money with a value of €100, handed out after completing the lifestyle program with the prerequisite that the participant had attended at least 75% of the appointments. The prerequisite for receiving the financial incentive was the most important attribute when patients had to decide whether or not to participate in a lifestyle program with an incentive, while the monetary value of the incentive had the lowest relative importance score.

The range of the potential uptake of all incentives was between 37.9% and 58.8%. This range is not very wide, taking into account the great variety of financial incentives that were examined in this study. Still, these differences in potential uptake do matter in practice, which makes this study relevant. It is a noticeable finding that the easiest requirement (registering for the lifestyle program and receiving the incentive at the start of the program) showed quite low potential uptake percentages (range between 37.9% and 45.4%). The study by Wanders et al. describes differences in effect size between out-of-pocket costs and financial rewards on the willingness to participate in a lifestyle program. In contrast to the results of our study, the study by Wanders et al. showed that a reward with a higher value is not always preferred [28], and that individuals may be offended by the high values of the incentive that were offered. In our study we used lower values for the incentive than the cut-off point of the study by Wanders et al., since a higher value than €100 was not feasible with a view to implementing the incentive in practice. Overall, the value did not have much impact on the potential uptake of the incentive (**Table 3 & Table 4**).

Sixty-two percent of the respondents have a household income between €1000 and €3000 per month. According to the OECD, the average household income in the Netherlands is about €2100 a month [29]. The average age of the respondents is 69.4 years, implying that most people are retired and entitled to a state pension and possibly to a supplementary pension scheme. In this group, it was found that the value of the financial incentive does not influence the potential uptake to a large extent. We hypothesize that retired individuals might not have very high costs, such as growing children or a mortgage, and may not need the money. The prerequisite for the financial incentive might be a more important determinant of their choice, because receiving the incentive and appreciating the reward is more justifiable if they have accomplished something.

Our target population consisted of patients with diabetes type 2 and/or cardiovascular disease. The average age was almost 70 years and half of the study population had a low level of education. In our study, 12.2% of the respondents had a low health literacy level. According to a report of the HLS-EU Consortium, about 29% of the Dutch population has an inadequate or problematic health literacy [30]. This relatively low percentage of individuals with low health literacy might be the result of selective response, since individuals with low health literacy might also not understand the questionnaire and therefore not respond. Completing a DCE is quite a complex task. One strength of our study is that the questionnaire was first pilot tested on readability and intelligibility, which is recommended in order to obtain valid results [31, 32]. By doing this, we reduced the chance that participants did not understand the final questionnaire. Furthermore, to limit the burden for the participants we divided the choice sets into two blocks.

There is little knowledge with regard to the response rates for DCE questionnaires. A study by Watson et al. found that the response rate decreases as the cognitive burden of the questionnaire increases [33]. The response rate in our DCE was 29.9%, which we believe is quite good, taking into account the aforementioned characteristics of our target population and the general complexity of the task. Overall, despite some limitations of the DCE technique, it is now the most accepted method to identify people’s preferences.

Overall, 64.2% of the respondents reported that a financial incentive would not motivate them to participate in and complete the lifestyle program. We sent this questionnaire to all patients with diabetes type 2 that are registered with a regional care group. This population also includes individuals who are sufficiently active. On the one hand, there might be selective non-response, with these active individuals not completing the questionnaire because they do not see the point of the program. On the other hand, the individuals who are sufficiently active and did fill out the questionnaire might not be motivated by receiving a financial incentive. If the respondents are not motivated by an incentive, does not mean that the wrong attributes were chosen in this study. The attributes are characteristics of the incentive that influence the choice for willing or not willing an incentive. We have chosen our attributes with input of our target population, so the selection of attributes was evidence based. Moreover, our results show a large heterogeneity in preferences. For example, the constant show that some respondent have a strong preference for receiving an incentive, whether others have a strong preference for not receiving an incentive. A similar pattern is seen for the value of the incentive. Some people attach importance to the value of the incentive, whether others do not. Due to the sample size, we were not able to specify the analyses, but it is likely that the heterogeneity could be explained partly by the respondents who state that an incentive would not motivate them.

Although just a small amount of research has been performed on the preferences of the target population for a financial incentive, it is becoming an increasingly important research area. Financial incentives may improve the effectiveness of, for example, prevention programs. One concern is that the implementation of financial incentives might pave the way for patients to misuse the available resources [34]. This might result in negative opinions and resistance from the public towards programs that contain financial incentives. In spite of the concerns that individuals may misuse HPFI, research shows that under certain conditions a HPFI is accepted more readily by the general public. These conditions are for example that the HPFI has to be effective and cost-effective and that the HPFI is closely monitored and evaluated [34–37].

Despite the arguments above, it is still useful to perform research on the preferences for and effectiveness of financial incentives. Lifestyle interventions can support good short-term adherence (up to twelve weeks) to exercise programs for chronically ill patients, but long-term adherence (up to four years) is poor and not well documented [38]. By completing lifestyle programs that are extended enough to achieve behavioral change, the chance that individuals will keep exercising in the long term might be higher. New and creative ways have to be found to increase the adherence of the chronically ill to lifestyle programs. Financial incentives might form one of these new instruments.

This study contributes to the knowledge of what chronically ill patients rate as more and less important with regard to financial incentives in lifestyle programs. The results of this DCE will be used in a study to evaluate the effectiveness of a financial incentive for improving the health of diabetes patients. By first identifying the preferred financial incentive, the probability that the financial incentive is effective will be maximized. In a broader perspective, this study contributes to the knowledge of preferences of individuals with regard to financial incentives.

## Conclusions

Among potential participants for a specified lifestyle program for the chronically ill, the most preferred financial incentive is cash money with a value of €100 that is handed out after the lifestyle program is finished with the prerequisite that the participant has attended at least 75% of the appointments. The potential uptake of the different financial incentives included in this DCE varied from 37.9% up to 58.8%. The value of the incentive did not significantly influence the potential uptake. However, the potential uptake and associated potential effect of the financial incentive is influenced by the type of financial incentive.

## Supporting information

S1 File Questionnaire translated in English. (DOCX)

S2 File Original questionnaire in Dutch. (DOCX)

## Abbreviations

DCE: discrete choice experiment
HPFI: health promoting financial incentive
