# Supplementary material for: Chronically ill patients’ preferences for a financial incentive in a lifestyle intervention. Results of a discrete choice experiment
Source: PLoS One. 2019 Jul 25;14(7):e0219112. doi: 10.1371/journal.pone.0219112 (PMC6657823; doi:10.1371/journal.pone.0219112)
Supplement: S1 File — (DOCX) [file pone.0219112.s001.docx]

**Vragenlijst** Financiële prikkels

**Toelichting bij de vragenlijst**

### Nowadays, much attention is paid to a healthy lifestyle.

### A healthy lifestyle is important to prevent diseases and other health problems. Many people struggle to maintain a healthy lifestyle or determine what is healthy for them. To help people to get a healthy lifestyle, lifestyle programs have been developed. It has been found that people find it easier to maintain a lifestyle program if they receive a reward for this. This questionnaire asks for your opinion towards lifestyle programs and financial incentives.

### The text blocks below briefly explain what we mean by these two terms.

*Lifestyle program*

A lifestyle program contains activities:

- that has the aim to work on your health.
- which focuses on both exercise and nutrition and
- that are offered by professional healthcare providers.

The program as referred to in this questionnaire has the following characteristics:

- Exercise sessions at the physiotherapist once a week for a period of 6 weeks.
- In about the same period 3 group sessions and 3 individual visits to the dietitian to get advice about a healthy diet.
- At the end of this period, together with you for a physical activity that suits you in the neighborhood.

### The questionnaire starts with a some general questions and a number of questions about your health.

### This is followed by a number of questions about lifestyle programs and financial incentives. Some questions require extra explanation.

### We ask you to consider the lifestyle program described above when completing the questionnaire.

This is followed by a number of questions about lifestyle programs and financial incentives. Some questions contain extra explanation.

Please complete the questionnaire as completely as possible. Every question can only have one answer option filled out, if otherwise it is mentioned. Filling out the questionnaire will take about 30 minutes. Your data will be treated confidential and will not be provided to others. The researcher will not be able to link the data to individual respondents.

**For questions about the questionnaire, please contact Claudia Molema, researcher.**

**E-mail: claudia.molema@rivm.nl**

**Phone number: 030-274 2753**

*Financial incentive*

With the term financial incentive we mean a reward with a monetary value. Such a financial incentive could for example, motivate people to start improving their lifestyle or achieving a certain result that has been agreed in advance.

| Please fill in the date on which you filled in the questionnaire. | **day month year**  **[ ][ ] [ ][ ] [ ][ ][ ][ ]** |
| --- | --- |
| 1. What is your age? | **[ ][ ] years** |
| 1. What is your gender? | - - Man   - Women |
| 1. What is your highest education? | - - Primary education (ISCED 1)   - Lower secondary education (ISCED 2)   - Upper secondary education (ISCED 3)   - Upper secondary work-related education (ISCED 3)   - Post-secondary but non-tertiary education (ISCED 4)   - First stage of tertiary education (ISCED 5 and 6)   - second stage of tertiary education (ISCED 7)   - Other, namely [ ] |
| 1. What is the average gross income of your household per month? | - - €1000 or less   - €1000 to €2000   - €2000 to €3000   - €3000 to €4000   - €4000 to €5000   - €5000 or more |
| 1. What is your marital status? | - - Single   - Living together   - Married |
| 1. How many persons does your household currently have? | - - 1 person   - 2 persons   - 3-4 persons   - 5 or more persons |

# **1** Below we ask you some questions about your personal situation

| 1. What is your country of birth? | - - The Netherlands   - Suriname   - Netherlands Antilles   - Aruba   - Turkey   - Morocco   - Other, namely **[** **]** |
| --- | --- |
| 1. What is the country of birth of your mother? | - - The Netherlands   - Suriname   - Netherlands Antilles   - Aruba   - Turkey   - Morocco   - Other, namely **[** **]** |
| 1. What is the country of birth of your father? | - - The Netherlands   - Suriname   - Netherlands Antilles   - Aruba   - Turkey   - Morocco   - Other, namely **[** **]** |
| 1. To which population group do you consider yourself to belong? *Please select only one answer.* | - - The Netherlands   - Suriname   - Netherlands Antilles   - Aruba   - Turkey   - Morocco   - Other, namely **[** **]** |

| 1. How many days a week do you cycle and/or walk on average per week?   *(this includes cycling and walking in your spare time, but also cycling or walking to, for example, work or the supermarket)* | - - 0 days   - 1 day   - 2 days   - 3 days   - 4 days   - 5 days   - 6 days   - 7 days | |
| --- | --- | --- |
| 1. How many time do you spend on cycling and walking on average per day?   *If your answer was ‘0 days’ for question 11, you can skip this question.* | - - Less than 15 minutes per day   - 15-30 minutes per day   - 30-60 minutes per day   - More than 60 minutes per day | |
| 1. Do you play sports?   *(walking and cycling are excluded)* | - - No, I do not play sports.   - Yes, I do play sports, but less than 1 time a week   - Ja, I do play sports for 1 -2 times a week   - Ja, ik sport 3 keer per week of vaker | |
| 1. How much time do you spend on exercise on average each session?   *If your answer was ‘No, I do not play sports’ for question 13, you can skip this question.* | - - Less than 15 minutes per session   - 30-60 minutes per session   - More than 60 minutes per session | |
| 1. Has something changed in the past three months in how much you exercise? | - - Yes, I started to move more   - Ja, I started to move less   - No, nothing has changed | |
| 1. How much fruit and vegetables do you eat on average per day? | *Fruit:*   - - 1 piece or less per day   - 2 pieces per day   - 3 pieces per day   - 4 pieces or more per day | *Vegetables:*   - - 1 serving spoon or less per day   - 2 serving spoons per day   - 3 serving spoons per day   - 4 serving spoons or more per day |
| 1. Has something changed in the past three months in how much fruits and vegetables you eat? | - - Yes, I started to eat more vegetables and/or fruit   - Yes, I started to eat less vegetables and/or fruit   - No, nothing has changed | |

| 1. What do you think about your own health status in general | - - Very good   - Good   - It’s ok   - Bad   - Very bad |
| --- | --- |
| ***> Would you mark the answer that fits best for your situation today.***  *Translation of questions below can be found on* [*www.euroqol.org*](http://www.euroqol.org) *EQ5D-5L questionnaire.* | |
| 1. Mobiliteit | - - Ik heb geen problemen met lopen   - Ik heb een beetje problemen met lopen   - Ik heb matige problemen met lopen   - Ik heb ernstige problemen met lopen   - Ik ben niet in staat om te lopen |
| 1. Zelfzorg | - - Ik heb geen problemen met mijzelf wassen of aankleden   - Ik heb een beetje problemen met mijzelf wassen of aankleden   - Ik heb matige problemen met mijzelf wassen of aankleden   - Ik heb ernstige problemen met mijzelf wassen of aankleden   - Ik ben niet in staat mijzelf te wassen of aan te kleden |
| 1. Dagelijkse activiteiten   *(bijv. werk, studie, huishouden, gezins- en*  *vrijetijdsactiviteiten)* | - - Ik heb geen problemen met mijn dagelijkse activiteiten   - Ik heb een beetje problemen met mijn dagelijkse activiteiten   - Ik heb matige problemen met mijn dagelijkse activiteiten   - Ik heb ernstige problemen met mijn dagelijkse activiteiten   - Ik ben niet in staat mijn dagelijkse activiteiten uit te voeren |
| 1. Pijn/ongemak | - - Ik heb geen pijn of ongemak   - Ik heb een beetje pijn of ongemak   - Ik heb matige pijn of ongemak   - Ik heb ernstige pijn of ongemak   - Ik heb extreme pijn of ongemak |
| 1. Angst/somberheid | - - Ik ben niet angstig of somber   - Ik ben een beetje angstig of somber   - Ik ben matig angstig of somber   - Ik ben erg angstig of somber   - Ik ben extreem angstig of somber |

| *Translation of questions 24, 25, and 26 can be found in the paper:*  Chew LD, Bradley KA, Boyko EJ. Brief questions to identify patients with inadequate health literacy. Family medicine. 2004;36(8):588-94. PubMed PMID: 15343421. | |
| --- | --- |
| 1. Hoe vaak helpt iemand u met het lezen van brieven of folders van uw huisarts of het ziekenhuis? | - - Nooit   - Af en toe   - Soms   - Vaak   - Altijd |
| 1. Hoe zeker bent u ervan dat u medische formulieren zelf goed invult? | - - Heel erg   - Nogal   - Een beetje   - Een klein beetje   - Helemaal niet |
| 1. Hoe vaak is het moeilijk voor u om meer te weten te komen over uw gezondheid, omdat u geschreven informatie niet goed begrijpt? | - - Nooit   - Af en toe   - Soms   - Vaak   - Altijd |
|  | |
| 1. Have you ever participated in a lifestyle program? | - - Yes, a lifestyle program for nutrition and/or exercise behavior.   - Yes, a lifestyle program for another subject than nutrition and/or exercise behavior   - No   - I don’t know |
| 1. Wat is uw mening over een leefstijlprogramma in het algemeen? | - - Very useful   - Useful   - Neutral   - Not very useful   - Not useful at all |
| 1. Zou u zelf graag (nog een keer) mee willen doen aan een leefstijlprogramma over voeding en/of bewegen? | - - Definetly yes   - Probably yes   - I don’t know   - Maybe   - Definetly no |

# **2** Which financial incentive do you prefer?

In this section of the questionnaire, we will present you multiple choice tasks between 2 situations. In total, we present you with 9 choice tasks. The purpose is that you always choose the situation that you would also choose in real life. The first column always shows the same, in the two columns next to it you will find small differences about the characteristics of the financial incentive.

Below is an explanation of the characteristics we use in this part of the questionnaire, followed by an example of a choice task.

***> It is important that you read first the explanation below before continuing with the questionnaire.***

**Form**: The reward can have several forms:
- Cash
- Voucher: this can be handed in at almost all larger stores.
- Dinner voucher: you can hand it in at participating restaurants.
- Theater and concert voucher: you can pay for tickets for a theater show or concert.

**Value**: The reward can have different values. The amount mentioned is the total amount, so if you receive a reward on several moments, then that is always a part of the total value:

- *15 euro*
- *35 euro*
- *65 euro*
- *100 euro*

**Moment:** The reward can be handed out at different moments

- *On beforehand*: you will receive the reward at the start of the lifestyle program
- *Afterwards:* you will receive the reward the end of the lifestyle program
- *Halfway and afterwards*: halfway of the lifestyle program you will receive half the reward

and after the program the other half of the reward.

**Prerequisite**: prerequisites can be set for getting the reward. If the prerequisite is not met, you will not receive the reward.

- *Registration for the lifestyle program:* if you register for the program, you will receive the reward.
- *75 % attendance at individual level individual:* you must have attended at least 75% of the meetings (i.e. at least 9 of the 12 meetings with the physiotherapist and the dietitian).
- *75 % attendance at group level:* the entire group must have attended at least 75% of the meetings (i.e. at least 9 out of 12 meetings with the physiotherapist and the dietitian).
- *Individual result fitness test:* you will receive the reward if you have a better score at the end of the program than at the start of the program on the fitness test. A fitness test measures your strength and condition.
- *Group result fitness tes*t: you receive the reward if at least 80% (8 out of 10) participants score better at the end of the program than at the start of the fitness test. A fitness test measures your strength and condition.

|  |  |
| --- | --- |
| 1. Which of the features described on the previous page is most important to you in choosing a financial incentive? | - - Form of the financial incentive   - Value of the financial incentive   - Moment of receiving the incentive   - Prerequisite for receiving the incentive |
| 1. Which of de characteristics described on the previous page is least important to you in choosing a financial incentive? | - - Form of the financial incentive   - Value of the financial incentive   - Moment of receiving the incentive   - Prerequisite for receiving the incentive |

**EXAMPLE**

| **Choice**  Imagine that your physician recommends that you participate in the lifestyle program as described above. Which financial incentive would motivate you most to participate in the lifestyle program and to complete it? | *Financial incentive A* | *Financial incentive B* |
| --- | --- | --- |
| **Form** | Cash money | Gift voucher |
| **Value** | 100 | 65 |
| **Moment** | Afterwards of the intervention | Afterwards of the intervention |
| **Prerequisite** | Group result fitness test | Individual result fitness test |

**⃞ ⃞**

If this incentive is offered to you in real-life in combination with a lifestyle program, would it motivate you to participate in the lifestyle program and to complete it?

- - Yes, the by me chosen financial incentive would motivate me to participate in the lifestyle program and to complete it, if this was offered to me in real-life
  - No, the by me chosen financial incentive would not motivate me to participate in the lifestyle program and to complete it, if this was offered to me in real-life.

Each choice set consists of two parts as you have seen above. First of all, you choose which of the 2 financial incentives are most appealing to you. Then there is a question whether the chosen incentvie would actually motivate you.

**It is important to make the choice between the two financial incentives in each situation first and then fill out the question that asks whether this would actually motivate you.**

There are now a number of choice sets, such as the example above. Please always tick the financial incentive you would prefer. When we mention the lifestyle program, we mean the program described on page 1.

**After the example and instruction, 9 choice tasks are presented. There are two versions, because 18 choice tasks were divided over two blocks.**

|  | |
| --- | --- |
| 1. What is your opinion about the use of financial inventives to motivate people to work on their health? | - - Very useful   - Useful   - Neutral   - Not very useful   - Not useful at all |
| 1. Do you think that a financial incentive can motivate people to work on their health? | - - Yes, I believe a financial incentive is a motivation for everyone   - Yes, but only for a small group of people   - No, I do not believe a financial incentive is a motivation |
| 1. Do you think that a financial incentive can motivate you to work on your health? | - - Yes   - No   - I don’t know |
| 1. What would you consider to be a realistic value of a financial incentive that you would receive in the already discussed lifestyle program? | - - **[ ]** euro |
| 1. If you receive a financial incentive from the combined lifestyle intervention discussed above, what form of reward would you prefer? | - - Cash money   - Voucher, for **[** **]**   - Present, namely **[** **]**   - Other, namely **[** **]** |
| 1. If you receive a financial incentive from the combined lifestyle intervention discussed above, what prerequisites do you think should be met before the reward is awarded? | - - No prerequisites   - Prerequisites in the form of **[** **]**   - Some prerequisite, but I don’t know in which form |
