# Supplementary material for: Chronically ill patients’ preferences for a financial incentive in a lifestyle intervention. Results of a discrete choice experiment
Source: PLoS One. 2019 Jul 25;14(7):e0219112. doi: 10.1371/journal.pone.0219112 (PMC6657823; doi:10.1371/journal.pone.0219112)
Supplement: S2 File — (DOCX) [file pone.0219112.s002.docx]

**Vragenlijst** Financiële prikkels

**Toelichting bij de vragenlijst**

### Er wordt tegenwoordig veel aandacht besteed aan een gezond leefpatroon. Gezond leven is belangrijk om ziekten en andere gezondheidsproblemen te voorkomen. Veel mensen hebben moeite om een gezonde leefstijl vol te houden of te bepalen wat gezond voor ze is. Om mensen op weg te helpen om een gezond leefpatroon te bereiken, zijn er leefstijlprogramma’s ontwikkeld. Het is gebleken dat mensen het makkelijker vinden om een leefstijlprogramma vol te houden als ze hiervoor een beloning krijgen. In deze vragenlijst wordt uw mening gevraagd over leefstijl- programma’s en financiële prikkels.

In onderstaande tekstblokken staat kort uitgelegd wat we met deze twee termen bedoelen.

*Leefstijlprogramma*

Met een leefstijlprogramma bedoelen we activiteiten:

- die als doel hebben te werken aan uw gezondheid,
- waarin aandacht is voor zowel bewegen als voeding en
- die door professionele zorgverleners worden aangeboden.

Het programma zoals bedoeld in deze vragenlijst ziet er als volgt uit:

- Gedurende een periode van 6 weken één keer per week naar de fysiotherapeut om te bewegen.
- In ongeveer dezelfde periode 3 groepssessies en 3 individuele bezoeken aan de diëtist om advies te krijgen over gezonde voeding.
- Aan het einde van deze periode wordt samen met u gezocht naar een beweegactiviteit in de buurt die bij u past.

### De vragenlijst start met enkele algemene vragen en een aantal vragen over uw gezondheid.

Daarna volgt een aantal vragen over leefstijlprogramma’s en financiële prikkels. Bij sommige vragen staat extra uitleg.

We willen u vragen om bij het invullen van de vragenlijst het leefstijlprogramma in gedachten te nemen dat hierboven is beschreven.

Vult u de vragenlijst alstublieft zo volledig mogelijk in. U kunt overal maar één antwoord invullen, tenzij anders is aangegeven. Het invullen van de vragenlijst zal ongeveer 30 minuten duren. Uw gegevens zullen vertrouwelijk worden behandeld en niet aan anderen worden verstrekt. Voor de onderzoeker zullen de gegevens niet naar u terug te leiden zijn.

**Voor vragen over de vragenlijst kunt u contact opnemen met Claudia Molema, onderzoeker.**

[**E-mail: claudia.molema@rivm.nl**](mailto:claudia.molema@rivm.nl) **Telefoonnummer: 030-274 2753**

*Financiële prikkel*

Met een financiële prikkel bedoelen we een beloning met een geldelijke waarde. Zo’n financiële prikkel zou

bijvoorbeeld kunnen helpen om mensen over te halen te beginnen met het verbeteren van hun leefstijl of bij het behalen van een bepaald resultaat wat vooraf is afgesproken.

# **1** Hieronder stellen we u enkele vragen over uw persoonlijke situatie

| Vult u hier alstublieft de datum in waarop u de vragenlijst heeft ingevuld. | **dag maand jaar**  **[ ][ ] [ ][ ] [ ][ ][ ][ ]** |
| --- | --- |
| 1. Wat is uw leeftijd? | **[ ][ ] jaar** |
| 1. Wat is uw geslacht? | - - Man   - Vrouw |
| 1. Wat is uw hoogst genoten opleiding? | - - Lager algemeen onderwijs (basisonderwijs)   - Lager beroepsonderwijs (LTS, LEAO)   - Middelbaar algemeen onderwijs (MAVO, MULO, VMBO)   - Middelbaar beroepsonderwijs (MTS, MEAO, MBO)   - Voortgezet algemeen onderwijs (HAVO, VWO, Atheneum, Gymnasium)   - Hoger beroepsonderwijs (HBO, HEAO, HTS)   - Wetenschappelijk onderwijs   - Anders, namelijk **[** **]** |
| 1. Wat is het gemiddelde bruto inkomen van uw huishouden per maand? | - - €1000 of minder   - €1000 tot €2000   - €2000 tot €3000   - €3000 tot €4000   - €4000 tot €5000   - €5000 of meer |
| 1. Wat is uw burgerlijke status? | - - Alleenstaand   - Samenwonend   - Gehuwd |
| 1. Uit hoeveel personen bestaat uw huishouden op dit moment? | - - 1 persoon   - 2 personen   - 3-4 personen   - 5 of meer personen |

| 1. Wat is uw geboorteland? | - - Nederland   - Suriname   - Nederlandse Antillen   - Aruba   - Turkije   - Marokko   - Overig, namelijk **[** **]** |
| --- | --- |
| 1. Wat is het geboorteland van uw moeder? | - - Nederland   - Suriname   - Nederlandse Antillen   - Aruba   - Turkije   - Marokko   - Overig, namelijk **[** **]** |
| 1. Wat is het geboorteland van uw vader? | - - Nederland   - Suriname   - Nederlandse Antillen   - Aruba   - Turkije   - Marokko   - Overig, namelijk **[** **]** |
| 1. Tot welke bevolkingsgroep rekent u zichzelf?   *Er is slechts één antwoord mogelijk.* | - - Nederlands   - Surinaams   - Antilliaans   - Arubaans   - Turks   - Marokkaans   - Anders, namelijk **[** **]** |

| 1. Hoeveel dagen fietst en/of wandelt u gemiddeld per week?   *(hier valt zowel fietsen en wandelen in uw vrije tijd onder, maar ook fietsen of wandelen naar bijvoorbeeld uw werk of de supermarkt)* | - - 0 dagen   - 1 dag   - 2 dagen   - 3 dagen   - 4 dagen   - 5 dagen   - 6 dagen   - 7 dagen | |
| --- | --- | --- |
| 1. Hoeveel tijd besteedt u hier gemiddeld per dag aan?   *Heeft u bij vraag 11 als antwoord ‘0 dagen’ gegeven, dan kunt u deze vraag overslaan.* | - - Minder dan 15 minuten per dag   - 15-30 minuten per dag   - 30-60 minuten per dag   - Meer dan 60 minuten per dag | |
| 1. Doet u aan sport? *(wandelen en fietsen worden hier niet meegerekend)* | - - Nee, ik doe niet aan sport.   - Ja, ik sport, maar minder dan 1 keer per week   - Ja, ik sport 1 -2 keer per week   - Ja, ik sport 3 keer per week of vaker | |
| 1. Hoeveel tijd besteedt u gemiddeld per keer aan het sporten?   *Heeft u bij vraag 13 als antwoord ‘Nee, ik doe niet aan sport’ gegeven, dan kunt u deze vraag overslaan*. | - - Minder dan 15 minuten per keer   - 15-30 minuten per keer   - 30-60 minuten per keer   - Meer dan 60 minuten per keer | |
| 1. Heeft u in de afgelopen 3 maanden iets veranderd aan hoeveel u beweegt? | - - Ja, ik ben meer gaan bewegen   - Ja, ik ben minder gaan bewegen   - Nee, er is niets veranderd | |
| 1. Hoeveel groente en fruit eet u gemiddeld per dag? | *Fruit:*   - - 1 stuk of minder per dag   - 2 stuks per dag   - 3 stuks per dag   - 4 stuks of meer per dag | *Groente:*   - - 1 opscheplepel of minder per dag   - 2 opscheplepels per dag   - 3 opscheplepels per dag   - 4 opscheplepels of meer per dag |
| 1. Heeft u in de afgelopen 3 maanden iets veranderd aan hoeveel groente en fruit u eet? | - - Ja, ik ben meer groente en/of fruit gaan eten   - Ja ik ben minder groente en/of fruit gaan eten   - Nee, er is niets veranderd | |

| 1. Wat vindt u in het algemeen van uw eigen gezondheid? | - - Zeer goed   - Goed   - Gaat wel   - Slecht   - Zeer slecht |
| --- | --- |
| ***> Wilt u hieronder steeds het antwoord aankruisen dat het best past bij u vandaag?*** | |
| 1. Mobiliteit | - - Ik heb geen problemen met lopen   - Ik heb een beetje problemen met lopen   - Ik heb matige problemen met lopen   - Ik heb ernstige problemen met lopen   - Ik ben niet in staat om te lopen |
| 1. Zelfzorg | - - Ik heb geen problemen met mijzelf wassen of aankleden   - Ik heb een beetje problemen met mijzelf wassen of aankleden   - Ik heb matige problemen met mijzelf wassen of aankleden   - Ik heb ernstige problemen met mijzelf wassen of aankleden   - Ik ben niet in staat mijzelf te wassen of aan te kleden |
| 1. Dagelijkse activiteiten   *(bijv. werk, studie, huishouden, gezins- en*  *vrijetijdsactiviteiten)* | - - Ik heb geen problemen met mijn dagelijkse activiteiten   - Ik heb een beetje problemen met mijn dagelijkse activiteiten   - Ik heb matige problemen met mijn dagelijkse activiteiten   - Ik heb ernstige problemen met mijn dagelijkse activiteiten   - Ik ben niet in staat mijn dagelijkse activiteiten uit te voeren |
| 1. Pijn/ongemak | - - Ik heb geen pijn of ongemak   - Ik heb een beetje pijn of ongemak   - Ik heb matige pijn of ongemak   - Ik heb ernstige pijn of ongemak   - Ik heb extreme pijn of ongemak |
| 1. Angst/somberheid | - - Ik ben niet angstig of somber   - Ik ben een beetje angstig of somber   - Ik ben matig angstig of somber   - Ik ben erg angstig of somber   - Ik ben extreem angstig of somber |

| ***> Informatie over gezondheid, ziekten of behandelingen kan soms ingewikkeld zijn. Wij zijn benieuwd naar uw ervaringen hiermee.*** | |
| --- | --- |
| 1. Hoe vaak helpt iemand u met het lezen van brieven of folders van uw huisarts of het ziekenhuis? | - - Nooit   - Af en toe   - Soms   - Vaak   - Altijd |
| 1. Hoe zeker bent u ervan dat u medische formulieren zelf goed invult? | - - Heel erg   - Nogal   - Een beetje   - Een klein beetje   - Helemaal niet |
| 1. Hoe vaak is het moeilijk voor u om meer te weten te komen over uw gezondheid, omdat u geschreven informatie niet goed begrijpt? | - - Nooit   - Af en toe   - Soms   - Vaak   - Altijd |
| ***> Hieronder volgen nog enkele vragen over uw ervaringen met en mening over leefstijlprogramma’s.*** | |
| 1. Heeft u ooit eerder aan een leefstijlprogramma mee gedaan? | - - Ja, een leefstijlprogramma over voeding en/of bewegen   - Ja, een leefstijlprogramma met een ander onderwerp dan voeding en/of bewegen   - Nee   - Weet ik niet |
| 1. Wat is uw mening over een leefstijlprogramma in het algemeen? | - - Zeer nuttig   - Nuttig   - Neutraal   - Niet zo nuttig   - Helemaal niet nuttig |
| 1. Zou u zelf graag (nog een keer) mee willen doen aan een leefstijlprogramma over voeding en/of bewegen? | - - Zeker wel   - Waarschijnlijk wel   - Weet ik niet   - Misschien   - Zeker niet |

# **2** Aan welke financiële prikkel geeft u de voorkeur?

In dit onderdeel van de vragenlijst leggen we u telkens een keuze voor tussen 2 situaties. In totaal leggen we u 9 keuzes voor. Het is de bedoeling dat u telkens de situatie kiest die u in het echte leven ook zou kiezen. In de eerste kolom staat steeds hetzelfde, in de twee kolommen ernaast vindt u kleine verschillen over de kenmerken van de financiële prikkel.

Hieronder wordt uitleg gegeven over de termen die we gebruiken in dit deel van de vragenlijst. Daarna volgt een voorbeeld van een keuze.

***> Het is belangrijk dat u dit eerst goed leest voordat u verder gaat met het invullen van de vragenlijst.***

**Vorm:** De beloning kan in meerdere vormen worden uitgereikt:

- *Contant geld*
- *VVV bon:* deze kunt u bij vrijwel alle grotere winkels inleveren.
- *Dinercheque:* deze kunt u bij deelnemende restaurants inleveren.
- *Theater- en concertbon:* hier kunt u kaartjes voor een theatershow of concert mee betalen.

**Hoogte:** De beloning kan verschillende hoogten hebben. Het genoemde bedrag is het totaalbedrag, dus als u op meerdere momenten een beloning krijgt, dan is dat steeds een deel van het totaalbedrag:

- *15 euro*
- *35 euro*
- *65 euro*
- *100 euro*

**Moment:** De beloning kan op verschillende momenten worden uitgereikt;

- *Vooraf:* u krijgt de beloning bij de start van het leefstijlprogramma
- *Achteraf:* u krijgt de beloning na afloop van het leefstijlprogramma
- *Halverwege en achteraf:* u krijgt halverwege het leefstijlprogramma de helft van de beloning

en na afloop van het programma de andere helft.

**Eisen:** er kunnen eisen gesteld worden aan het krijgen van de beloning. Wanneer niet wordt voldaan aan de gestelde eis, dan zult u geen beloning ontvangen.

- *Inschrijven voor leefstijlprogramma individu:* als u zich inschrijft voor het programma, dan krijgt u de beloning.
- *75 % aanwezigheid individu:* u moet zelf minimaal 75% van de bijeenkomsten deelgenomen hebben (dus minimaal 9 van de 12 bijeenkomsten met de fysiotherapeut en de diëtist).
- *75 % aanwezigheid groep:* de gehele groep moet minimaal 75% van de bijeenkomsten deelgenomen hebben (dus minimaal 9 van de 12 bijeenkomsten met de fysiotherapeut en de diëtist).
- *Prestatie fitheidstest individu:* u ontvangt de beloning als u aan het einde van het programma een betere score hebt dan aan het begin van het programma op de fitheidstest. Een fitheidstest meet uw kracht en conditie.
- *Prestatie fitheidstest groep:* u ontvangt de beloning als tenminste 80% (8 van de 10) deelnemers aan het einde van het programma beter scoren dan bij het begin op de fitheidstest. Een fitheidstest meet uw kracht en conditie.

|  |  |
| --- | --- |
| 1. Welke van de op de vorige pagina beschreven kenmerken is voor u het meest belangrijk in de keuze voor een financiële prikkel? | - - Vorm van de financiële prikkel   - Hoogte van de financiële prikkel   - Moment waarop de financiële prikkel wordt uitgereikt aan u   - Eisen welke er gesteld worden voordat u de financiële prikkel krijgt |
| 1. Welke van de op de vorige pagina beschreven kenmerken is voor u het minst belangrijk in de keuze voor een financiële prikkel? | - - Vorm van de financiële prikkel   - Hoogte van de financiële prikkel   - Moment waarop de financiële prikkel wordt uitgereikt aan u   - Eisen welke er gesteld worden voordat u de financiële prikkel krijgt |

| **Voorbeeld**  Stelt u zich voor dat uw huisarts u aanraadt om deel te nemen aan het leefstijlprogramma dat is beschreven op de eerste pagina van de vragenlijst. Welke financiële prikkel zou u dan het meeste motiveren om aan het leefstijlprogramma deel te nemen EN deze af te maken? | *Financiële prikkel A* | *Financiële prikkel B* |
| --- | --- | --- |
| **Vorm** | Dinercheque | VVV bon |
| **Hoogte** | 65 | 35 |
| **Moment** | Achteraf | Vooraf |
| **Eisen** | 75% aanwezigheid groep | Inschrijven leefstijlprogramma |

**⊠ ⃞**

Elke keuzeset bestaat uit twee delen zoals u hierboven hebt kunnen zien. Allereerst kiest u welke van de 2 financiële prikkels u het meest aanspreekt in een situatie waarbij u moet kiezen. Daarna volgt er een vraag of de gekozen prikkel u in werkelijkheid ook zou motiveren.

**Het is belangrijk om bij elke situatie eerst de keuze tussen de 2 financiële prikkels te maken en pas daarna de vraag in te vullen waarin wordt gevraagd of dit u in werkelijkheid ook zou motiveren.**

Er volgen nu een aantal keuzesets zoals het voorbeeld hierboven. Wilt u telkens de financiële prikkel aankruisen die u het liefst zou willen. Wanneer we het over het leefstijlprogramma hebben, dan bedoelen we het programma wat op pagina 1 is beschreven.

*In dit geval heeft u gekozen voor optie A. Dit betekent dat u een financiële prikkel ontvangt in de vorm van een dinercheque ter waarde van 65 euro. Deze krijgt u alleen als de groep gemiddeld 75% van alle consulten aanwezig is geweest en u krijgt de beloning achteraf. Daarnaast geeft u aan dat wanneer deze prikkel u in werkelijkheid wordt aangeboden, u niet gemotiveerd bent om deel te nemen aan het leefstijlprogramma en ook niet om deze af te maken.*

Als u in werkelijkheid deze prikkel aangeboden krijgt bij een leefstijlprogramma, zou dit u dan motiveren om aan het leefstijlprogramma deel te nemen en deze ook af te maken?

- - Ja, de door mij gekozen financiële prikkel zou mij wel motiveren om deel te nemen aan het leefstijlprogramma en deze ook af te maken als deze mij in werkelijkheid wordt aangeboden.
- Nee, de door mij gekozen financiële prikkel zou mij niet motiveren om deel te nemen aan het leefstijlprogramma en ook niet om het programma af te maken als deze mij in werkelijkheid wordt aangeboden.

| **Keuze 1**  Stelt u zich voor dat uw huisarts u aanraadt om deel te nemen aan het leefstijlprogramma dat is beschreven op de eerste pagina van de vragenlijst. Welke financiële prikkel zou u dan het meeste motiveren om aan het leefstijlprogramma deel te nemen EN deze af te maken? | *Financiële prikkel A* | *Financiële prikkel B* |
| --- | --- | --- |
| **Vorm** | Contant geld | VVV bon |
| **Hoogte** | 100 | 65 |
| **Moment** | Achteraf | Achteraf |
| **Eisen** | Groepsprestatie fitheidstest | Individuele prestatie fitheidstest |

**⃞ ⃞**

Als u in werkelijkheid deze prikkel aangeboden krijgt bij een leefstijlprogramma, zou dit u dan motiveren om aan het leefstijlprogramma deel te nemen en deze ook af te maken?

- - Ja, de door mij gekozen financiële prikkel zou mij wel motiveren om deel te nemen aan het leefstijlprogramma en deze ook af te maken als deze mij in werkelijkheid wordt aangeboden.
  - Nee, de door mij gekozen financiële prikkel zou mij niet motiveren om deel te nemen aan het leefstijlprogramma en ook niet om het programma af te maken als deze mij in werkelijkheid wordt aangeboden.

| **Keuze 2**  Stelt u zich voor dat uw huisarts u aanraadt om deel te nemen aan het leefstijlprogramma dat is beschreven op de eerste pagina van de vragenlijst. Welke financiële prikkel zou u dan het meeste motiveren om aan het leefstijlprogramma deel te nemen EN deze af te maken? | *Financiële prikkel A* | *Financiële prikkel B* |
| --- | --- | --- |
| **Vorm** | Dinercheque | Contant geld |
| **Hoogte** | 65 | 15 |
| **Moment** | Vooraf | Achteraf |
| **Eisen** | Inschrijven leefstijlprogramma | Individuele prestatie fitheidstest |

**⃞ ⃞**

Als u in werkelijkheid deze prikkel aangeboden krijgt bij een leefstijlprogramma, zou dit u dan motiveren om aan het leefstijlprogramma deel te nemen en deze ook af te maken?

- - Ja, de door mij gekozen financiële prikkel zou mij wel motiveren om deel te nemen aan het leefstijlprogramma en deze ook af te maken als deze mij in werkelijkheid wordt aangeboden.
  - Nee, de door mij gekozen financiële prikkel zou mij niet motiveren om deel te nemen aan het leefstijlprogramma en ook niet om het programma af te maken als deze mij in werkelijkheid wordt aangeboden.

| **Keuze 3**  Stelt u zich voor dat uw huisarts u aanraadt om deel te nemen aan het leefstijlprogramma dat is beschreven op de eerste pagina van de vragenlijst. Welke financiële prikkel zou u dan het meeste motiveren om aan het leefstijlprogramma deel te nemen EN deze af te maken? | *Financiële prikkel A* | *Financiële prikkel B* |
| --- | --- | --- |
| **Vorm** | Dinercheque | Theater- en concertbon |
| **Hoogte** | 100 | 35 |
| **Moment** | Achteraf | Vooraf |
| **Eisen** | Individuele prestatie fitheidstest | Inschrijven leefstijlprogramma |

**⃞ ⃞**

Als u in werkelijkheid deze prikkel aangeboden krijgt bij een leefstijlprogramma, zou dit u dan motiveren om aan het leefstijlprogramma deel te nemen en deze ook af te maken?

- - Ja, de door mij gekozen financiële prikkel zou mij wel motiveren om deel te nemen aan het leefstijlprogramma en deze ook af te maken als deze mij in werkelijkheid wordt aangeboden.
  - Nee, de door mij gekozen financiële prikkel zou mij niet motiveren om deel te nemen aan het leefstijlprogramma en ook niet om het programma af te maken als deze mij in werkelijkheid wordt aangeboden.

| **Keuze 4**  Stelt u zich voor dat uw huisarts u aanraadt om deel te nemen aan het leefstijlprogramma dat is beschreven op de eerste pagina van de vragenlijst. Welke financiële prikkel zou u dan het meeste motiveren om aan het leefstijlprogramma deel te nemen EN deze af te maken? | *Financiële prikkel A* | *Financiële prikkel B* |
| --- | --- | --- |
| **Vorm** | Contant geld | VVV bon |
| **Hoogte** | 15 | 15 |
| **Moment** | Achteraf | Halverwege en achteraf |
| **Eisen** | 75% aanwezigheid individu | 75% aanwezigheid groep |

**⃞ ⃞**

Als u in werkelijkheid deze prikkel aangeboden krijgt bij een leefstijlprogramma, zou dit u dan motiveren om aan het leefstijlprogramma deel te nemen en deze ook af te maken?

- - Ja, de door mij gekozen financiële prikkel zou mij wel motiveren om deel te nemen aan het leefstijlprogramma en deze ook af te maken als deze mij in werkelijkheid wordt aangeboden.
  - Nee, de door mij gekozen financiële prikkel zou mij niet motiveren om deel te nemen aan het leefstijlprogramma en ook niet om het programma af te maken als deze mij in werkelijkheid wordt aangeboden.

| **Keuze 5**  Stelt u zich voor dat uw huisarts u aanraadt om deel te nemen aan het leefstijlprogramma dat is beschreven op de eerste pagina van de vragenlijst. Welke financiële prikkel zou u dan het meeste motiveren om aan het leefstijlprogramma deel te nemen EN deze af te maken? | *Financiële prikkel A* | *Financiële prikkel B* |
| --- | --- | --- |
| **Vorm** | Theater- en concertbon | Dinercheque |
| **Hoogte** | 65 | 65 |
| **Moment** | Halverwege en achteraf | Achteraf |
| **Eisen** | 75% aanwezigheid groep | Inschrijven leefstijlprogramma |

**⃞ ⃞**

Als u in werkelijkheid deze prikkel aangeboden krijgt bij een leefstijlprogramma, zou dit u dan motiveren om aan het leefstijlprogramma deel te nemen en deze ook af te maken?

- - Ja, de door mij gekozen financiële prikkel zou mij wel motiveren om deel te nemen aan het leefstijlprogramma en deze ook af te maken als deze mij in werkelijkheid wordt aangeboden.
  - Nee, de door mij gekozen financiële prikkel zou mij niet motiveren om deel te nemen aan het leefstijlprogramma en ook niet om het programma af te maken als deze mij in werkelijkheid wordt aangeboden.

| **Keuze 6**  Stelt u zich voor dat uw huisarts u aanraadt om deel te nemen aan het leefstijlprogramma dat is beschreven op de eerste pagina van de vragenlijst. Welke financiële prikkel zou u dan het meeste motiveren om aan het leefstijlprogramma deel te nemen EN deze af te maken? | *Financiële prikkel A* | *Financiële prikkel B* |
| --- | --- | --- |
| **Vorm** | Theater- en concertbon | VVV bon |
| **Hoogte** | 35 | 35 |
| **Moment** | Achteraf | Achteraf |
| **Eisen** | Inschrijven leefstijlprogramma | 75% aanwezigheid individu |

**⃞ ⃞**

Als u in werkelijkheid deze prikkel aangeboden krijgt bij een leefstijlprogramma, zou dit u dan motiveren om aan het leefstijlprogramma deel te nemen en deze ook af te maken?

- - Ja, de door mij gekozen financiële prikkel zou mij wel motiveren om deel te nemen aan het leefstijlprogramma en deze ook af te maken als deze mij in werkelijkheid wordt aangeboden.
  - Nee, de door mij gekozen financiële prikkel zou mij niet motiveren om deel te nemen aan het leefstijlprogramma en ook niet om het programma af te maken als deze mij in werkelijkheid wordt aangeboden.

| **Keuze 7**  Stelt u zich voor dat uw huisarts u aanraadt om deel te nemen aan het leefstijlprogramma dat is beschreven op de eerste pagina van de vragenlijst. Welke financiële prikkel zou u dan het meeste motiveren om aan het leefstijlprogramma deel te nemen EN deze af te maken? | *Financiële prikkel A* | *Financiële prikkel B* |
| --- | --- | --- |
| **Vorm** | VVV bon | Dinercheque |
| **Hoogte** | 15 | 35 |
| **Moment** | Achteraf | Achteraf |
| **Eisen** | Inschrijven leefstijlprogramma | Individuele prestatie fitheidstest |

**⃞ ⃞**

Als u in werkelijkheid deze prikkel aangeboden krijgt bij een leefstijlprogramma, zou dit u dan motiveren om aan het leefstijlprogramma deel te nemen en deze ook af te maken?

- - Ja, de door mij gekozen financiële prikkel zou mij wel motiveren om deel te nemen aan het leefstijlprogramma en deze ook af te maken als deze mij in werkelijkheid wordt aangeboden.
  - Nee, de door mij gekozen financiële prikkel zou mij niet motiveren om deel te nemen aan het leefstijlprogramma en ook niet om het programma af te maken als deze mij in werkelijkheid wordt aangeboden.

| **Keuze 8**  Stelt u zich voor dat uw huisarts u aanraadt om deel te nemen aan het leefstijlprogramma dat is beschreven op de eerste pagina van de vragenlijst. Welke financiële prikkel zou u dan het meeste motiveren om aan het leefstijlprogramma deel te nemen EN deze af te maken? | *Financiële prikkel A* | *Financiële prikkel B* |
| --- | --- | --- |
| **Vorm** | Theater- en concertbon | Contant geld |
| **Hoogte** | 65 | 100 |
| **Moment** | Achteraf | Halverwege en achteraf |
| **Eisen** | Individuele prestatie fitheidstest | 75% aanwezigheid individu |

**⃞ ⃞**

Als u in werkelijkheid deze prikkel aangeboden krijgt bij een leefstijlprogramma, zou dit u dan motiveren om aan het leefstijlprogramma deel te nemen en deze ook af te maken?

- - Ja, de door mij gekozen financiële prikkel zou mij wel motiveren om deel te nemen aan het leefstijlprogramma en deze ook af te maken als deze mij in werkelijkheid wordt aangeboden.
  - Nee, de door mij gekozen financiële prikkel zou mij niet motiveren om deel te nemen aan het leefstijlprogramma en ook niet om het programma af te maken als deze mij in werkelijkheid wordt aangeboden.

| **Keuze 9**  Stelt u zich voor dat uw huisarts u aanraadt om deel te nemen aan het leefstijlprogramma dat is beschreven op de eerste pagina van de vragenlijst. Welke financiële prikkel zou u dan het meeste motiveren om aan het leefstijlprogramma deel te nemen EN deze af te maken? | *Financiële prikkel A* | *Financiële prikkel B* |
| --- | --- | --- |
| **Vorm** | Contant geld | Theater- en concertbon |
| **Hoogte** | 35 | 100 |
| **Moment** | Halverwege en achteraf | Achteraf |
| **Eisen** | 75% aanwezigheid groep | 75% aanwezigheid individu |

**⃞ ⃞**

Als u in werkelijkheid deze prikkel aangeboden krijgt bij een leefstijlprogramma, zou dit u dan motiveren om aan het leefstijlprogramma deel te nemen en deze ook af te maken?

- - Ja, de door mij gekozen financiële prikkel zou mij wel motiveren om deel te nemen aan het leefstijlprogramma en deze ook af te maken als deze mij in werkelijkheid wordt aangeboden.
  - Nee, de door mij gekozen financiële prikkel zou mij niet motiveren om deel te nemen aan het leefstijlprogramma en ook niet om het programma af te maken als deze mij in werkelijkheid wordt aangeboden.

| ***> Tot slot volgen nu nog enkele vragen over financiële prikkels.*** | |
| --- | --- |
| 1. Wat is uw mening over het inzetten van financiële prikkels om mensen te motiveren aan hun gezondheid te werken? | - - Zeer nuttig   - Nuttig   - Neutraal   - Niet zo nuttig   - Helemaal niet nuttig |
| 1. Denkt u dat een financiële prikkel mensen kan motiveren om aan hun gezondheid te werken? | - - Ja, ik denk dat dit een motivatie is voor iedereen.   - Ja, maar alleen voor kleine groepen mensen.   - Nee, ik denk dat het voor niemand een motivatie zal zijn. |
| 1. Denkt u dat een financiële prikkel u kan motiveren om aan uw gezondheid te werken? | - - Ja   - Nee   - Weet ik niet |
| 1. Wat zou u een reëel waarde vinden van een financiële prikkel welke u zou krijgen bij het al besproken leefstijlprogramma? | - - **[ ]** euro |
| 1. Als u een financiële prikkel aangeboden krijgt bij de al besproken gecombineerde leefstijlinterventie, wat voor soort beloning zou u dan het liefst krijgen? | - - Contant geld   - Cadeaubon, voor **[** **]**   - Cadeau, namelijk **[** **]**   - Anders, namelijk **[** **]** |
| 1. Als u een financiële prikkel aangeboden krijgt bij de al besproken gecombineerde leefstijlinterventie, aan welke eisen vindt u dat moeten worden voldaan voordat de beloning wordt uitgereikt? | - - Geen eisen   - Wel eisen, namelijk **[** **]**   - Wel eisen, maar ik weet niet welke eisen |

*> Dit is het einde van de vragenlijst, wilt u controleren of u alle vragen heeft ingevuld?*

Bedankt voor uw deelname!

*> U kunt de vragenlijst terug sturen in de bijgevoegde antwoordenveloppe*
